# Methyl 3′-(2,5-di­methyl­benz­yl)-1′-methyl-2-oxo-4′-phenyl­spiro­[indoline-3,2′-pyrrolidine]-3′-carboxyl­ate chloro­form monosolvate

**DOI:** 10.1107/S1600536814004073

**Published:** 2014-02-28

**Authors:** S. Karthikeyan, P. Narayanan, K. Sethusankar, Anthonisamy Devaraj, Manickam Bakthadoss

**Affiliations:** a Department of Physics, RKM Vivekananda College (Autonomous), Chennai 600 004, India; b Department of Organic Chemistry, University of Madras, Maraimalai Campus, Chennai 600 025, India

## Abstract

In the title solvate, C_29_H_30_N_2_O_3_·CHCl_3_, the dihedral angle between the indole ring system (r.m.s. deviation = 0.050 Å) and the 4-methyl­pyrrolidine ring is 88.88 (8)°. The latter ring adopts an envelope conformation with the N atom as the flap. Its mean plane makes dihedral angles of 86.94 (11) and 42.08 (9)° with the phenyl and di­methyl­benzene rings, respectively. The mol­ecular conformation is stabilized by intra­molecular C—H⋯O hydrogen bonds, which generate *S*(6) and *S*(9) ring motifs. The chloro­form solvent mol­ecule is linked to the organic mol­ecule by a C—H⋯O hydrogen bond involving the carbonyl O atom of the carboxyl­ate group. In the crystal, mol­ecules are linked *via* bifurcated N—H⋯(N,O) and C—H⋯O hydrogen bonds, forming chains propagating along [001].

## Related literature   

For the biological activity of spiro compounds and oxindole derivatives, see: Bhattacharya *et al.* (1982 ▶); Chande *et al.* (2005 ▶); Glover *et al.* (1998 ▶). For a related crystal structure, see: Karthikeyan *et al.* (2014 ▶). For puckering parameters, see: Cremer & Pople (1975 ▶). For graph-set motif notation, see: Bernstein *et al.* (1995 ▶). For bond-length distortions in small rings, see: Allen (1981 ▶).
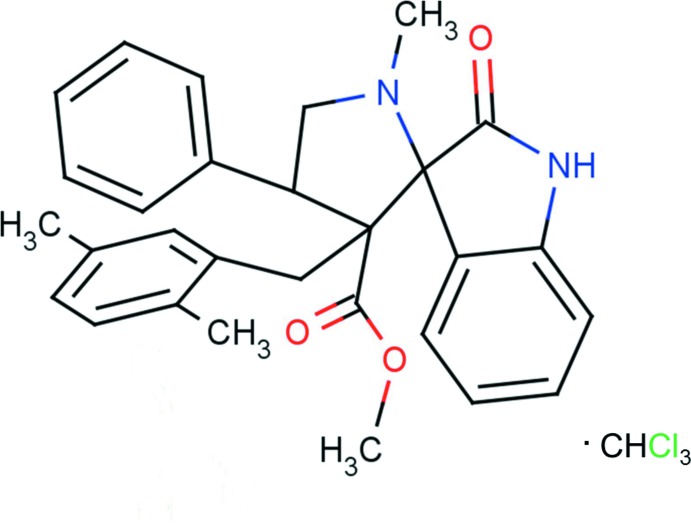


## Experimental   

### 

#### Crystal data  

C_29_H_30_N_2_O_3_·CHCl_3_
*M* *_r_* = 573.92
Monoclinic, 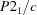

*a* = 12.9164 (4) Å
*b* = 17.6167 (5) Å
*c* = 12.4548 (5) Å
β = 98.135 (2)°
*V* = 2805.50 (16) Å^3^
*Z* = 4
Mo *K*α radiation
μ = 0.36 mm^−1^
*T* = 293 K
0.35 × 0.30 × 0.25 mm

#### Data collection  

Bruker Kappa APEXII CCD diffractometer
32563 measured reflections
8093 independent reflections
4986 reflections with *I* > 2σ(*I*)
*R*_int_ = 0.032

#### Refinement  

*R*[*F* ^2^ > 2σ(*F* ^2^)] = 0.052
*wR*(*F* ^2^) = 0.161
*S* = 1.01
8093 reflections
347 parameters
H-atom parameters constrained
Δρ_max_ = 0.38 e Å^−3^
Δρ_min_ = −0.46 e Å^−3^

### 

Data collection: *APEX2* (Bruker, 2008 ▶); cell refinement: *SAINT* (Bruker, 2008 ▶); data reduction: *SAINT*; program(s) used to solve structure: *SHELXS97* (Sheldrick, 2008 ▶); program(s) used to refine structure: *SHELXL97* (Sheldrick, 2008 ▶); molecular graphics: *ORTEP-3 for Windows* (Farrugia, 2012 ▶) and *Mercury* (Macrae *et al.*, 2008 ▶); software used to prepare material for publication: *SHELXL97* and *PLATON* (Spek, 2009 ▶).

## Supplementary Material

Crystal structure: contains datablock(s) global, I. DOI: 10.1107/S1600536814004073/su2703sup1.cif

Structure factors: contains datablock(s) I. DOI: 10.1107/S1600536814004073/su2703Isup2.hkl

Supporting information file. DOI: 10.1107/S1600536814004073/su2703Isup3.cml

CCDC reference: 988124

Additional supporting information:  crystallographic information; 3D view; checkCIF report

## Figures and Tables

**Table 1:** Hydrogen-bond geometry (Å, °)

*D*—H⋯*A*	*D*—H	H⋯*A*	*D*⋯*A*	*D*—H⋯*A*
C5—H5⋯O1	0.93	2.45	3.292 (2)	151
C18—H18*B*⋯O1	0.97	2.52	3.127 (2)	120
C30—H30⋯O2	0.98	2.47	3.319 (3)	144
N2—H2*A*⋯O2^i^	0.86	2.65	3.252 (2)	128
N2—H2*A*⋯N1^i^	0.86	2.20	2.936 (2)	143
C7—H7⋯O1^ii^	0.98	2.57	3.359 (2)	138

Symmetry codes: (i) 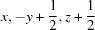; (ii) 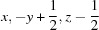.

**Fig. 1.**
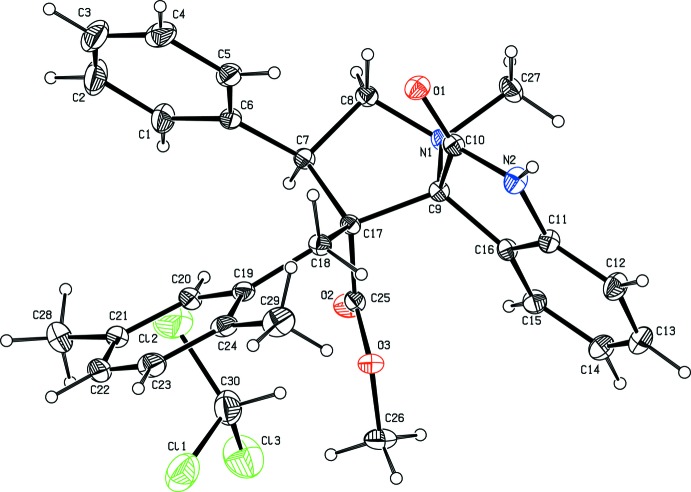
The molecular structure of the title compound, with atom labelling. Displacement ellipsoids are drawn at the 30% probability level.

**Fig. 2.**
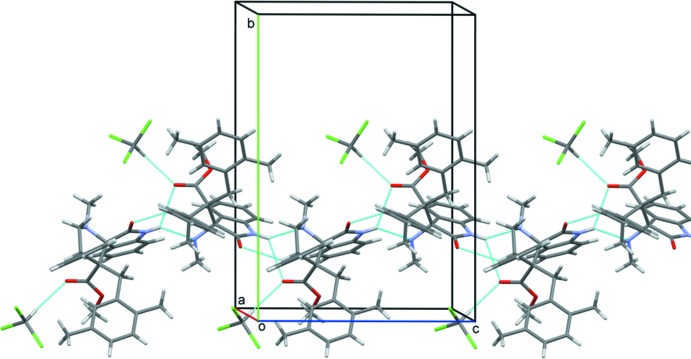
A partial view along the a axis of the crystal packing of the title compound. The N—H···N, N-H···O and C—H···O hydrogen bonds are shown as dashed lines (see Table 1 for details).

## supplementary crystallographic information

### 1. Comment

Synthesis of spiro compounds has drawn considerable attention from chemists, in view of their very good antimycobacterial activity (Chande *et al.*, 2005). Oxindole derivatives are known to be potent inhibitors of monoamine oxidase (MAO) in human urine and rat tissues (Glover *et al.*, 1998) and potent antagonists of *in vitro* receptor binding by atrial natriuretic peptide besides possessing a wide range of central nervous system activities (Bhattacharya *et al.*, 1982).

The molecular structure of the title compound is illustrated in Fig 1. In the molecule, there is C—H···O hydrogen bond, forming an *S*(6) and *S*(9) ring motif (Bernstein *et al.*, 1995). The indole ring system is essentially planar with a maximum deviation of 0.0782 (17) Å for the atom C10. The mean plane of the indole ring system forms dihedral angle of 88.88 (8)° with central mean plane of pyrrolidine five membered ring. The latter forms a dihedral angle of 42.08 (10)° with the benzyl ring. Atom O1 significantly deviates from the mean plane of the indole ring system by 0.1965 (12) Å. The molecular dimensions in the title compound are in excellent agreement with the those reported for a related compound (Karthikeyan *et al.*, 2014).

The spiro-pyrrolidine ring adopts an envelope conformation with atom N1 at the flap position. The distance to the flap position from the mean plane of the spiro carbon is 0.2628 (15) Å. The puckering parameters (Cremer & Pople, 1975) of the ring are Q_2_ = 0.4155 (17) Å and φ_2_ = 0.5 (2)°. The central spiro-pyrrolidine ring is perpendicular to the phenyl ring (C1—C6) with a dihedral angle of 86.94 (11)°. The carbonyl group and the benzyl ring have an (+)anti-periplanar conformation with the torsion angle (C18—C17—C25—O2) of 152.45 (17)°.

In the benzene ring (C11—C16) of the indole ring system, the expansion of the ipso angles at C11, C13 and C14 [122.20 (17), 120.82 (19) and 120.86 (19)°, respectively] and contraction of the apical angles at C12, C15 and C16 [117.80 (19), 118.99 (18) and 119.24 (16)°, respectively] are caused by the fusion of the smaller pyrrole ring to the six-membered benzene ring and the strain is taken up by the angular distortion rather than by bond-length distortions (Allen, 1981). The carboxyl group and oxindole ring system are (-)*syn*-clinal to each other with the torsion angle (C9—C17—C25—O2) of -87.22 (9)°.

In the crystal, (Fig. 2 and Table 1), molecules are linked by N2—H2A···N1^(i)^ [*x*,-*y* + 1/2,*z* + 1/2] and N2—H2A···O2^(i)^ [*x*,-*y* + 1/2,*z* + 1/2] bifurcated hydrogen bonds which together generate C_2_^1^[*R*_2_^1^(6)] chains (Bernstein *et al.*, 1995), running parallel to [001]. The intermolecular C7—H7···O1^(ii)^ [*x*,-*y* + 1/2,*z* - 1/2] interaction forms *C*(6) chains running parallel to the same [001] axis. The CHCl_3_ solvent molecule is involved in an intramolecular hydrogen bond with the C═ O atom, O2, of the carboxylate group.

### 2. Experimental

A mixture of (*E*)-methyl 2-(2,5-dimethylbenzyl)-3-phenylacrylate (2 mmol), isatin (2 mmol) and sarcosine (2 mmol) in acetonitrile (8 ml) was refluxed for 12 h. After completion of the reaction, as indicated by TLC, the mixture was concentrated. The resulting crude mass was diluted with water (10 ml) and extracted with ethyl acetate (3 × 10 ml). The combined organic layers were washed with brine (2 × 10 ml) and dried over anhydrous Na_2_SO_4_. The organic layer was concentrated and the residue purified by column chromatography on silica gel (Acme 100–200 mesh), using ethyl acetate:hexanes (2:8) to afford the title compound as a colourless crystals in 67% yield.

### 3. Refinement

The H atoms could all be located in difference electron-density maps. In the final cycles of refinement they were treated as riding atoms and their distances were geometrically constrained: C—H = 0.93 and 0.96 Å for CH and CH_3_H atoms, respectively, with *U*_iso_(H) = 1.5 *U*_eq_(C– methyl) and = 1.2*U*_eq_(C) for other H atoms.

### Crystal data

**Table:**

C_29_H_30_N_2_O_3_·CHCl_3_	*F*(000) = 1200
*M**_r_* = 573.92	*D*_x_ = 1.359 Mg m^−^^3^
Monoclinic, *P*2_1_/*c*	Mo *K*α radiation, λ = 0.71073 Å
Hall symbol: -P 2ybc	Cell parameters from 8093 reflections
*a* = 12.9164 (4) Å	θ = 2.0–30.0°
*b* = 17.6167 (5) Å	µ = 0.36 mm^−^^1^
*c* = 12.4548 (5) Å	*T* = 293 K
β = 98.135 (2)°	Block, colourless
*V* = 2805.50 (16) Å^3^	0.35 × 0.30 × 0.25 mm
*Z* = 4	

### Data collection

**Table:**

Bruker Kappa APEXII CCD diffractometer	4986 reflections with *I* > 2σ(*I*)
Radiation source: fine-focus sealed tube	*R*_int_ = 0.032
Graphite monochromator	θ_max_ = 30.0°, θ_min_ = 2.0°
ω scans	*h* = −18→15
32563 measured reflections	*k* = −24→17
8093 independent reflections	*l* = −16→17

### Refinement

**Table:**

Refinement on *F*^2^	Primary atom site location: structure-invariant direct methods
Least-squares matrix: full	Secondary atom site location: difference Fourier map
*R*[*F*^2^ > 2σ(*F*^2^)] = 0.052	Hydrogen site location: inferred from neighbouring sites
*wR*(*F*^2^) = 0.161	H-atom parameters constrained
*S* = 1.01	*w* = 1/[σ^2^(*F*_o_^2^) + (0.0743*P*)^2^ + 0.8771*P*] where *P* = (*F*_o_^2^ + 2*F*_c_^2^)/3
8093 reflections	(Δ/σ)_max_ = 0.029
347 parameters	Δρ_max_ = 0.38 e Å^−^^3^
0 restraints	Δρ_min_ = −0.46 e Å^−^^3^

### Special details

**Table:**

Geometry. All e.s.d.'s (except the e.s.d. in the dihedral angle between two l.s. planes) are estimated using the full covariance matrix. The cell e.s.d.'s are taken into account individually in the estimation of e.s.d.'s in distances, angles and torsion angles; correlations between e.s.d.'s in cell parameters are only used when they are defined by crystal symmetry. An approximate (isotropic) treatment of cell e.s.d.'s is used for estimating e.s.d.'s involving l.s. planes.
Refinement. Refinement of *F*^2^ against ALL reflections. The weighted *R*-factor *wR* and goodness of fit *S* are based on *F*^2^, conventional *R*-factors *R* are based on *F*, with *F* set to zero for negative *F*^2^. The threshold expression of *F*^2^ > σ(*F*^2^) is used only for calculating *R*-factors(gt) *etc*. and is not relevant to the choice of reflections for refinement. *R*-factors based on *F*^2^ are statistically about twice as large as those based on *F*, and *R*- factors based on ALL data will be even larger.

### Fractional atomic coordinates and isotropic or equivalent isotropic displacement parameters (Å^2^)

**Table:**

	*x*	*y*	*z*	*U*_iso_*/*U*_eq_	
C1	0.52972 (18)	0.16392 (14)	0.1611 (2)	0.0606 (6)	
H1	0.5616	0.1446	0.1045	0.073*	
C2	0.4214 (2)	0.16690 (18)	0.1514 (3)	0.0870 (10)	
H2	0.3813	0.1492	0.0884	0.104*	
C3	0.3732 (2)	0.19554 (17)	0.2334 (3)	0.0844 (10)	
H3	0.3006	0.1969	0.2265	0.101*	
C4	0.43157 (18)	0.22214 (14)	0.3255 (3)	0.0670 (7)	
H4	0.3987	0.2417	0.3813	0.080*	
C5	0.54030 (15)	0.22008 (11)	0.33620 (19)	0.0473 (5)	
H5	0.5796	0.2393	0.3986	0.057*	
C6	0.59068 (14)	0.18961 (10)	0.25467 (16)	0.0391 (4)	
C7	0.70826 (13)	0.19113 (9)	0.25662 (13)	0.0300 (3)	
H7	0.7220	0.1678	0.1886	0.036*	
C8	0.74566 (13)	0.27309 (9)	0.25403 (14)	0.0319 (3)	
H8A	0.7355	0.2928	0.1805	0.038*	
H8B	0.7086	0.3053	0.2991	0.038*	
C9	0.86380 (12)	0.21922 (9)	0.39155 (12)	0.0269 (3)	
C10	0.82724 (13)	0.25825 (9)	0.49252 (13)	0.0309 (3)	
C11	0.99571 (13)	0.21963 (10)	0.54390 (14)	0.0347 (4)	
C12	1.09263 (16)	0.20958 (12)	0.60490 (17)	0.0482 (5)	
H12	1.1043	0.2224	0.6780	0.058*	
C13	1.17168 (16)	0.17983 (13)	0.5537 (2)	0.0540 (5)	
H13	1.2374	0.1715	0.5932	0.065*	
C14	1.15450 (15)	0.16239 (12)	0.44510 (19)	0.0497 (5)	
H14	1.2091	0.1434	0.4117	0.060*	
C15	1.05653 (14)	0.17275 (10)	0.38447 (16)	0.0391 (4)	
H15	1.0456	0.1616	0.3108	0.047*	
C16	0.97590 (13)	0.19984 (9)	0.43528 (13)	0.0301 (3)	
C17	0.78538 (12)	0.15185 (9)	0.35030 (12)	0.0272 (3)	
C18	0.73755 (13)	0.11476 (9)	0.44370 (14)	0.0324 (4)	
H18A	0.7936	0.1058	0.5028	0.039*	
H18B	0.6903	0.1511	0.4693	0.039*	
C19	0.67848 (13)	0.04089 (9)	0.42034 (15)	0.0356 (4)	
C20	0.64281 (14)	0.01636 (10)	0.31604 (17)	0.0418 (4)	
H20	0.6602	0.0446	0.2581	0.050*	
C21	0.58202 (15)	−0.04865 (11)	0.2939 (2)	0.0506 (5)	
C22	0.56029 (16)	−0.09077 (12)	0.3815 (2)	0.0592 (6)	
H22	0.5208	−0.1349	0.3698	0.071*	
C23	0.59634 (16)	−0.06826 (12)	0.4854 (2)	0.0573 (6)	
H23	0.5806	−0.0978	0.5429	0.069*	
C24	0.65561 (14)	−0.00280 (11)	0.50774 (18)	0.0443 (5)	
C25	0.85308 (13)	0.09330 (9)	0.30117 (14)	0.0314 (3)	
C26	0.96441 (19)	−0.01230 (12)	0.3421 (2)	0.0563 (6)	
H26A	0.9259	−0.0453	0.2896	0.084*	
H26B	0.9945	−0.0414	0.4039	0.084*	
H26C	1.0191	0.0123	0.3103	0.084*	
C27	0.90549 (16)	0.34317 (11)	0.31638 (17)	0.0454 (5)	
H27A	0.8690	0.3715	0.3651	0.068*	
H27B	0.9024	0.3701	0.2490	0.068*	
H27C	0.9772	0.3368	0.3479	0.068*	
C28	0.5391 (2)	−0.06896 (15)	0.1793 (2)	0.0740 (8)	
H28A	0.4752	−0.0969	0.1785	0.111*	
H28B	0.5891	−0.0995	0.1487	0.111*	
H28C	0.5256	−0.0234	0.1373	0.111*	
C29	0.69211 (19)	0.01897 (14)	0.6230 (2)	0.0604 (6)	
H29A	0.6638	−0.0158	0.6706	0.091*	
H29B	0.6689	0.0695	0.6358	0.091*	
H29C	0.7671	0.0172	0.6366	0.091*	
C30	0.8351 (2)	−0.03717 (16)	0.0148 (2)	0.0727 (7)	
H30	0.8775	−0.0035	0.0660	0.087*	
N1	0.85668 (11)	0.26899 (7)	0.29684 (11)	0.0297 (3)	
N2	0.90658 (11)	0.25256 (9)	0.57607 (12)	0.0391 (4)	
H2A	0.9025	0.2674	0.6411	0.047*	
O1	0.74367 (10)	0.28839 (7)	0.49619 (10)	0.0406 (3)	
O2	0.87103 (11)	0.09300 (8)	0.20917 (10)	0.0470 (3)	
O3	0.89518 (10)	0.04398 (7)	0.37572 (10)	0.0376 (3)	
Cl1	0.80296 (6)	−0.11741 (5)	0.08605 (8)	0.0992 (3)	
Cl2	0.72435 (8)	0.01165 (5)	−0.03976 (10)	0.1107 (3)	
Cl3	0.91063 (9)	−0.06436 (6)	−0.08484 (8)	0.1116 (3)	

### Atomic displacement parameters (Å^2^)

**Table:**

	*U*^11^	*U*^22^	*U*^33^	*U*^12^	*U*^13^	*U*^23^
C1	0.0494 (12)	0.0582 (13)	0.0670 (15)	−0.0072 (10)	−0.0171 (11)	0.0041 (11)
C2	0.0539 (16)	0.086 (2)	0.108 (3)	−0.0188 (15)	−0.0341 (16)	0.0200 (18)
C3	0.0336 (12)	0.0785 (19)	0.137 (3)	−0.0047 (12)	−0.0028 (16)	0.0423 (19)
C4	0.0418 (12)	0.0548 (13)	0.108 (2)	0.0071 (10)	0.0222 (13)	0.0316 (14)
C5	0.0376 (10)	0.0399 (10)	0.0651 (14)	0.0012 (8)	0.0102 (9)	0.0142 (9)
C6	0.0340 (9)	0.0296 (8)	0.0517 (11)	−0.0008 (7)	−0.0013 (8)	0.0098 (8)
C7	0.0339 (8)	0.0287 (8)	0.0267 (8)	−0.0002 (6)	0.0018 (6)	0.0003 (6)
C8	0.0367 (9)	0.0299 (8)	0.0287 (8)	0.0003 (7)	0.0039 (7)	0.0051 (6)
C9	0.0298 (8)	0.0286 (7)	0.0228 (7)	−0.0004 (6)	0.0055 (6)	−0.0013 (6)
C10	0.0341 (9)	0.0312 (8)	0.0283 (8)	−0.0014 (7)	0.0072 (7)	−0.0031 (6)
C11	0.0317 (8)	0.0382 (9)	0.0341 (9)	−0.0009 (7)	0.0044 (7)	−0.0018 (7)
C12	0.0415 (11)	0.0599 (13)	0.0399 (11)	0.0017 (9)	−0.0056 (8)	−0.0038 (9)
C13	0.0344 (10)	0.0611 (13)	0.0632 (14)	0.0037 (9)	−0.0046 (9)	−0.0019 (11)
C14	0.0335 (10)	0.0517 (12)	0.0653 (14)	0.0056 (8)	0.0119 (9)	−0.0041 (10)
C15	0.0374 (9)	0.0410 (10)	0.0402 (10)	0.0003 (8)	0.0100 (8)	−0.0042 (8)
C16	0.0306 (8)	0.0298 (8)	0.0301 (8)	−0.0030 (6)	0.0055 (6)	−0.0002 (6)
C17	0.0314 (8)	0.0250 (7)	0.0253 (8)	−0.0013 (6)	0.0046 (6)	0.0003 (6)
C18	0.0356 (9)	0.0304 (8)	0.0324 (9)	−0.0007 (7)	0.0090 (7)	0.0043 (6)
C19	0.0292 (8)	0.0284 (8)	0.0502 (11)	0.0026 (6)	0.0093 (7)	0.0089 (7)
C20	0.0380 (10)	0.0307 (9)	0.0562 (12)	−0.0020 (7)	0.0049 (8)	0.0039 (8)
C21	0.0340 (10)	0.0314 (9)	0.0848 (16)	0.0004 (8)	0.0029 (10)	−0.0025 (10)
C22	0.0367 (10)	0.0332 (10)	0.107 (2)	−0.0054 (8)	0.0098 (12)	0.0091 (12)
C23	0.0420 (11)	0.0420 (11)	0.0911 (19)	0.0000 (9)	0.0208 (12)	0.0279 (11)
C24	0.0349 (9)	0.0375 (9)	0.0637 (13)	0.0059 (8)	0.0178 (9)	0.0191 (9)
C25	0.0345 (8)	0.0276 (8)	0.0318 (9)	−0.0022 (7)	0.0036 (7)	−0.0021 (6)
C26	0.0637 (14)	0.0415 (11)	0.0639 (14)	0.0206 (10)	0.0101 (11)	−0.0004 (10)
C27	0.0527 (11)	0.0331 (9)	0.0501 (12)	−0.0124 (8)	0.0055 (9)	0.0033 (8)
C28	0.0675 (16)	0.0518 (14)	0.097 (2)	−0.0107 (12)	−0.0083 (14)	−0.0168 (13)
C29	0.0594 (14)	0.0661 (14)	0.0595 (14)	0.0025 (11)	0.0219 (11)	0.0265 (11)
C30	0.0636 (16)	0.0764 (17)	0.0758 (18)	−0.0125 (13)	0.0022 (13)	−0.0162 (14)
N1	0.0347 (7)	0.0274 (7)	0.0272 (7)	−0.0048 (5)	0.0050 (5)	0.0023 (5)
N2	0.0374 (8)	0.0554 (9)	0.0243 (7)	0.0019 (7)	0.0037 (6)	−0.0096 (6)
O1	0.0380 (7)	0.0472 (7)	0.0373 (7)	0.0077 (6)	0.0084 (5)	−0.0086 (5)
O2	0.0607 (9)	0.0490 (8)	0.0330 (7)	0.0132 (7)	0.0124 (6)	−0.0031 (6)
O3	0.0426 (7)	0.0309 (6)	0.0394 (7)	0.0085 (5)	0.0064 (5)	0.0036 (5)
Cl1	0.0716 (5)	0.1114 (6)	0.1077 (7)	−0.0031 (4)	−0.0107 (4)	0.0367 (5)
Cl2	0.0970 (6)	0.0791 (5)	0.1532 (9)	0.0107 (4)	0.0078 (6)	0.0290 (5)
Cl3	0.1246 (8)	0.1139 (7)	0.1039 (7)	−0.0108 (6)	0.0420 (6)	−0.0332 (5)

### Geometric parameters (Å, º)

**Table:**

C1—C6	1.386 (3)	C17—C18	1.538 (2)
C1—C2	1.388 (4)	C18—C19	1.516 (2)
C1—H1	0.9300	C18—H18A	0.9700
C2—C3	1.366 (5)	C18—H18B	0.9700
C2—H2	0.9300	C19—C20	1.385 (3)
C3—C4	1.363 (5)	C19—C24	1.398 (3)
C3—H3	0.9300	C20—C21	1.394 (3)
C4—C5	1.392 (3)	C20—H20	0.9300
C4—H4	0.9300	C21—C22	1.380 (3)
C5—C6	1.389 (3)	C21—C28	1.500 (4)
C5—H5	0.9300	C22—C23	1.371 (4)
C6—C7	1.516 (2)	C22—H22	0.9300
C7—C8	1.524 (2)	C23—C24	1.390 (3)
C7—C17	1.582 (2)	C23—H23	0.9300
C7—H7	0.9800	C24—C29	1.497 (3)
C8—N1	1.459 (2)	C25—O2	1.201 (2)
C8—H8A	0.9700	C25—O3	1.330 (2)
C8—H8B	0.9700	C26—O3	1.437 (2)
C9—N1	1.462 (2)	C26—H26A	0.9600
C9—C16	1.512 (2)	C26—H26B	0.9600
C9—C10	1.564 (2)	C26—H26C	0.9600
C9—C17	1.597 (2)	C27—N1	1.456 (2)
C10—O1	1.210 (2)	C27—H27A	0.9600
C10—N2	1.357 (2)	C27—H27B	0.9600
C11—C12	1.381 (3)	C27—H27C	0.9600
C11—C16	1.385 (2)	C28—H28A	0.9600
C11—N2	1.397 (2)	C28—H28B	0.9600
C12—C13	1.382 (3)	C28—H28C	0.9600
C12—H12	0.9300	C29—H29A	0.9600
C13—C14	1.374 (3)	C29—H29B	0.9600
C13—H13	0.9300	C29—H29C	0.9600
C14—C15	1.391 (3)	C30—Cl2	1.724 (3)
C14—H14	0.9300	C30—Cl1	1.750 (3)
C15—C16	1.378 (2)	C30—Cl3	1.751 (3)
C15—H15	0.9300	C30—H30	0.9800
C17—C25	1.534 (2)	N2—H2A	0.8600
			
C6—C1—C2	120.4 (3)	C19—C18—H18A	107.9
C6—C1—H1	119.8	C17—C18—H18A	107.9
C2—C1—H1	119.8	C19—C18—H18B	107.9
C3—C2—C1	120.6 (3)	C17—C18—H18B	107.9
C3—C2—H2	119.7	H18A—C18—H18B	107.2
C1—C2—H2	119.7	C20—C19—C24	118.66 (17)
C4—C3—C2	120.0 (2)	C20—C19—C18	122.66 (16)
C4—C3—H3	120.0	C24—C19—C18	118.63 (17)
C2—C3—H3	120.0	C19—C20—C21	123.04 (19)
C3—C4—C5	120.1 (3)	C19—C20—H20	118.5
C3—C4—H4	120.0	C21—C20—H20	118.5
C5—C4—H4	120.0	C22—C21—C20	117.2 (2)
C6—C5—C4	120.7 (2)	C22—C21—C28	122.3 (2)
C6—C5—H5	119.6	C20—C21—C28	120.4 (2)
C4—C5—H5	119.6	C23—C22—C21	120.71 (19)
C1—C6—C5	118.11 (19)	C23—C22—H22	119.6
C1—C6—C7	117.89 (19)	C21—C22—H22	119.6
C5—C6—C7	123.60 (17)	C22—C23—C24	122.2 (2)
C6—C7—C8	109.65 (14)	C22—C23—H23	118.9
C6—C7—C17	121.93 (14)	C24—C23—H23	118.9
C8—C7—C17	105.16 (13)	C23—C24—C19	118.2 (2)
C6—C7—H7	106.4	C23—C24—C29	119.59 (19)
C8—C7—H7	106.4	C19—C24—C29	122.23 (18)
C17—C7—H7	106.4	O2—C25—O3	123.23 (16)
N1—C8—C7	104.09 (13)	O2—C25—C17	125.55 (15)
N1—C8—H8A	110.9	O3—C25—C17	111.12 (14)
C7—C8—H8A	110.9	O3—C26—H26A	109.5
N1—C8—H8B	110.9	O3—C26—H26B	109.5
C7—C8—H8B	110.9	H26A—C26—H26B	109.5
H8A—C8—H8B	109.0	O3—C26—H26C	109.5
N1—C9—C16	111.94 (13)	H26A—C26—H26C	109.5
N1—C9—C10	113.17 (13)	H26B—C26—H26C	109.5
C16—C9—C10	101.09 (12)	N1—C27—H27A	109.5
N1—C9—C17	102.79 (12)	N1—C27—H27B	109.5
C16—C9—C17	118.65 (13)	H27A—C27—H27B	109.5
C10—C9—C17	109.60 (12)	N1—C27—H27C	109.5
O1—C10—N2	125.77 (16)	H27A—C27—H27C	109.5
O1—C10—C9	126.59 (15)	H27B—C27—H27C	109.5
N2—C10—C9	107.64 (14)	C21—C28—H28A	109.5
C12—C11—C16	122.20 (17)	C21—C28—H28B	109.5
C12—C11—N2	127.95 (17)	H28A—C28—H28B	109.5
C16—C11—N2	109.79 (15)	C21—C28—H28C	109.5
C11—C12—C13	117.80 (19)	H28A—C28—H28C	109.5
C11—C12—H12	121.1	H28B—C28—H28C	109.5
C13—C12—H12	121.1	C24—C29—H29A	109.5
C14—C13—C12	120.82 (19)	C24—C29—H29B	109.5
C14—C13—H13	119.6	H29A—C29—H29B	109.5
C12—C13—H13	119.6	C24—C29—H29C	109.5
C13—C14—C15	120.86 (19)	H29A—C29—H29C	109.5
C13—C14—H14	119.6	H29B—C29—H29C	109.5
C15—C14—H14	119.6	Cl2—C30—Cl1	111.11 (15)
C16—C15—C14	118.99 (18)	Cl2—C30—Cl3	111.84 (17)
C16—C15—H15	120.5	Cl1—C30—Cl3	109.54 (16)
C14—C15—H15	120.5	Cl2—C30—H30	108.1
C15—C16—C11	119.24 (16)	Cl1—C30—H30	108.1
C15—C16—C9	131.30 (15)	Cl3—C30—H30	108.1
C11—C16—C9	109.27 (14)	C27—N1—C8	113.35 (14)
C25—C17—C18	109.19 (13)	C27—N1—C9	115.28 (14)
C25—C17—C7	109.56 (13)	C8—N1—C9	105.71 (12)
C18—C17—C7	117.73 (13)	C10—N2—C11	111.97 (14)
C25—C17—C9	104.86 (12)	C10—N2—H2A	124.0
C18—C17—C9	112.13 (13)	C11—N2—H2A	124.0
C7—C17—C9	102.51 (12)	C25—O3—C26	117.19 (15)
C19—C18—C17	117.69 (15)		
			
C6—C1—C2—C3	0.4 (4)	C10—C9—C17—C25	−149.46 (13)
C1—C2—C3—C4	0.6 (4)	N1—C9—C17—C18	−151.71 (13)
C2—C3—C4—C5	−0.1 (4)	C16—C9—C17—C18	84.19 (17)
C3—C4—C5—C6	−1.4 (3)	C10—C9—C17—C18	−31.11 (17)
C2—C1—C6—C5	−1.8 (3)	N1—C9—C17—C7	−24.50 (14)
C2—C1—C6—C7	−174.9 (2)	C16—C9—C17—C7	−148.60 (14)
C4—C5—C6—C1	2.3 (3)	C10—C9—C17—C7	96.10 (14)
C4—C5—C6—C7	174.93 (18)	C25—C17—C18—C19	−52.72 (19)
C1—C6—C7—C8	109.52 (19)	C7—C17—C18—C19	72.97 (19)
C5—C6—C7—C8	−63.2 (2)	C9—C17—C18—C19	−168.48 (14)
C1—C6—C7—C17	−127.14 (19)	C17—C18—C19—C20	−17.2 (2)
C5—C6—C7—C17	60.2 (2)	C17—C18—C19—C24	165.28 (15)
C6—C7—C8—N1	159.17 (14)	C24—C19—C20—C21	2.2 (3)
C17—C7—C8—N1	26.43 (16)	C18—C19—C20—C21	−175.26 (17)
N1—C9—C10—O1	55.2 (2)	C19—C20—C21—C22	−2.1 (3)
C16—C9—C10—O1	175.02 (17)	C19—C20—C21—C28	175.4 (2)
C17—C9—C10—O1	−58.9 (2)	C20—C21—C22—C23	0.9 (3)
N1—C9—C10—N2	−124.66 (15)	C28—C21—C22—C23	−176.5 (2)
C16—C9—C10—N2	−4.79 (17)	C21—C22—C23—C24	0.1 (3)
C17—C9—C10—N2	121.26 (15)	C22—C23—C24—C19	0.0 (3)
C16—C11—C12—C13	1.1 (3)	C22—C23—C24—C29	179.6 (2)
N2—C11—C12—C13	−175.89 (19)	C20—C19—C24—C23	−1.1 (3)
C11—C12—C13—C14	1.3 (3)	C18—C19—C24—C23	176.45 (16)
C12—C13—C14—C15	−1.3 (3)	C20—C19—C24—C29	179.27 (18)
C13—C14—C15—C16	−1.0 (3)	C18—C19—C24—C29	−3.1 (3)
C14—C15—C16—C11	3.2 (3)	C18—C17—C25—O2	152.45 (17)
C14—C15—C16—C9	177.68 (17)	C7—C17—C25—O2	22.2 (2)
C12—C11—C16—C15	−3.3 (3)	C9—C17—C25—O2	−87.22 (19)
N2—C11—C16—C15	174.10 (16)	C18—C17—C25—O3	−30.95 (18)
C12—C11—C16—C9	−178.93 (17)	C7—C17—C25—O3	−161.22 (13)
N2—C11—C16—C9	−1.49 (19)	C9—C17—C25—O3	89.38 (15)
N1—C9—C16—C15	−50.4 (2)	C7—C8—N1—C27	−171.60 (14)
C10—C9—C16—C15	−171.15 (18)	C7—C8—N1—C9	−44.41 (16)
C17—C9—C16—C15	69.1 (2)	C16—C9—N1—C27	−62.53 (18)
N1—C9—C16—C11	124.47 (15)	C10—C9—N1—C27	50.92 (19)
C10—C9—C16—C11	3.73 (16)	C17—C9—N1—C27	169.04 (14)
C17—C9—C16—C11	−116.06 (15)	C16—C9—N1—C8	171.44 (13)
C6—C7—C17—C25	122.61 (16)	C10—C9—N1—C8	−75.11 (15)
C8—C7—C17—C25	−111.99 (14)	C17—C9—N1—C8	43.01 (15)
C6—C7—C17—C18	−2.9 (2)	O1—C10—N2—C11	−175.49 (17)
C8—C7—C17—C18	122.51 (15)	C9—C10—N2—C11	4.3 (2)
C6—C7—C17—C9	−126.44 (15)	C12—C11—N2—C10	175.34 (19)
C8—C7—C17—C9	−1.03 (16)	C16—C11—N2—C10	−1.9 (2)
N1—C9—C17—C25	89.93 (14)	O2—C25—O3—C26	−0.7 (3)
C16—C9—C17—C25	−34.17 (17)	C17—C25—O3—C26	−177.36 (16)

### Hydrogen-bond geometry (Å, º)

**Table:**

*D*—H···*A*	*D*—H	H···*A*	*D*···*A*	*D*—H···*A*
C5—H5···O1	0.93	2.45	3.292 (2)	151
C18—H18*B*···O1	0.97	2.52	3.127 (2)	120
C30—H30···O2	0.98	2.47	3.319 (3)	144
N2—H2*A*···O2^i^	0.86	2.65	3.252 (2)	128
N2—H2*A*···N1^i^	0.86	2.20	2.936 (2)	143
C7—H7···O1^ii^	0.98	2.57	3.359 (2)	138

Symmetry codes: (i) *x*, −*y*+1/2, *z*+1/2; (ii) *x*, −*y*+1/2, *z*−1/2.
